# rs1004819 Is the Main Disease-Associated *IL23R* Variant in German Crohn's Disease Patients: Combined Analysis of *IL23R*, *CARD15,* and *OCTN1/2* Variants

**DOI:** 10.1371/journal.pone.0000819

**Published:** 2007-09-05

**Authors:** Jürgen Glas, Julia Seiderer, Martin Wetzke, Astrid Konrad, Helga-Paula Török, Silke Schmechel, Laurian Tonenchi, Christine Grassl, Julia Dambacher, Simone Pfennig, Kerstin Maier, Thomas Griga, Wolfram Klein, Jörg T. Epplen, Uwe Schiemann, Christian Folwaczny, Peter Lohse, Burkhard Göke, Thomas Ochsenkühn, Bertram Müller-Myhsok, Matthias Folwaczny, Thomas Mussack, Stephan Brand

**Affiliations:** 1 Department of Medicine II - Grosshadern, University of Munich, Munich, Germany; 2 Clinic for Preventive Dentistry and Parodontology, University of Munich, Munich, Germany; 3 Department of Surgery, University of Munich, Munich, Germany; 4 Department of Internal Medicine, Knappschaftskrankenhaus Dortmund, Dortmund, Germany; 5 Department of Human Genetics, Ruhr-University Bochum, Bochum, Germany; 6 Department of General Internal Medicine, Inselspital Bern, Bern, Switzerland; 7 Institute of Clinical Chemistry, Grosshadern, University of Munich, Munich, Germany; 8 Max Planck Institute of Psychiatry, Munich, Germany; Innsbruck Medical University, Austria

## Abstract

**Background:**

The *IL23R* gene has been identified as a susceptibility gene for inflammatory bowel disease (IBD) in the North American population. The aim of our study was to test this association in a large German IBD cohort and to elucidate potential interactions with other IBD genes as well as phenotypic consequences of *IL23R* variants.

**Methods:**

Genomic DNA from 2670 Caucasian individuals including 833 patients with Crohn's disease (CD), 456 patients with ulcerative colitis (UC), and 1381 healthy unrelated controls was analyzed for 10 *IL23R* SNPs. Genotyping included the NOD2 variants p.Arg702Trp, p.Gly908Arg, and p.Leu1007fsX1008 and polymorphisms in *SLC22A4*/OCTN1 (1672 C→T) and *SLC22A5*/OCTN2 (–207 G→C).

**Results:**

All *IL23R* gene variants analyzed displayed highly significant associations with CD. The strongest association was found for the SNP rs1004819 [*P* = 1.92×10^−11^; OR 1.56; 95 % CI (1.37–1.78)]. 93.2% of the rs1004819 TT homozygous carriers as compared to 78% of CC wildtype carriers had ileal involvement [*P* = 0.004; OR 4.24; CI (1.46–12.34)]. The coding SNP rs11209026 (p.Arg381Gln) was protective for CD [*P* = 8.04×10^−8^; OR 0.43; CI (0.31–0.59)]. Similar, but weaker associations were found in UC. There was no evidence for epistasis between the *IL23R* gene and the CD susceptibility genes *CARD15* and *SLC22A4/5*.

**Conclusion:**

*IL23R* is an IBD susceptibility gene, but has no epistatic interaction with *CARD15* and *SLC22A4/5*. rs1004819 is the major *IL23R* variant associated with CD in the German population, while the p.Arg381Gln *IL23R* variant is a protective marker for CD and UC.

## Introduction

Inflammatory bowel diseases (IBD) such as Crohn's disease (CD) and ulcerative colitis (UC) are chronic inflammatory disorders of the gastrointestinal tract characterized by a perpetuated mucosal immune response which is triggered by intestinal bacteria in genetically susceptible individuals [1], [2]. Since the identification of *CARD15* (encoding NOD2) as the first susceptibility gene for CD in 2001 [3], [4], several variants of proteins involved in bacterial recognition were demonstrated to be associated with IBD or certain IBD phenotypes [5], [6], [7], [8], [9], [10], [11].

Most recently, Duerr et al. [12] identified the *IL23R* gene located on chromosome 1p31 as an IBD susceptibility gene in a genome-wide association study testing 308,332 single nucleotide polymorphisms (SNPs) in 547 non-Jewish ileal CD cases and 548 controls. Subsequent analyses by the same group confirmed these findings in an independent Jewish CD cohort and in 883 nuclear families [12]. In this study, the uncommon *IL23R* coding variant rs11209026 (1142G>A; p.Arg381Gln) was associated with strong protection against CD [12]. This was recently confirmed in a British cohort [13] and for pediatric CD [14], [15]. For nine other variants located within the *IL23R* gene or in the intergenic area with the adjacent *IL12RB2* gene, Duerr et al. [12] demonstrated associations with CD with p-values of <0.0001. Some of these variants also showed weaker associations with UC [12].

IL-23, a member of the IL-12 cytokine family, represents a proinflammatory cytokine and is composed of the IL-23-specific p19 subunit and a p40 subunit, which is also part of the IL-12 heterodimer. IL-23, mainly produced by activated macrophages and dendritic cells (DCs), activates Th17 cells which differentiate under the influence of the cytokines IL-6 and TGF-β [16]. Very recent studies indicate also a role for IL-21 in this process [17], [18], [19]. In Th17 cells, the transcription factor RORγt activates the expression of proinflammatory Th17 cytokines such as IL-17A, IL-17F and IL-22. In contrast, IL-12 induces the differentiation of naive CD4+ T cells into IFN-γ producing Th1 cells through activation of STAT4. IFN-γ induced signals are transduced by STAT1, which activates a downstream transcription factor, T-bet, which enhances the expression of genes specific to Th1 cells. Th1 cells promote a cell-mediated immune response to intracellular pathogens, while IL-4-, IL-5- and IL-13-producing Th2 cells promote a humoral immune response to extracellular pathogens. Similarly, there is increasing evidence for the IL-23/IL-17 axis as a key pathway in the development of chronic inflammation and in host defense against bacterial infection, promoting local tissue inflammation by stimulating T cells, macrophages and fibroblasts as well as endothelial and epithelial cells to produce proinflammatory cytokines, metalloproteinases, and chemokines (reviewed in Ref.[20]). Recent studies demonstrated a critical role for the IL-23/IL-17 pathway in T-cell mediated colitis in different mouse models [21], [22], [23], in tumour immunity as demonstrated in human tumor tissues and cancer cell lines as well as in knockout mice [24], [25], and in the development of autoimmune diseases including autoimmune encephalomyelitis in a mouse model [26] and in patients with multiple sclerosis [27]. A very recent study demonstrated that monoclonal antibodies (mAb) directed against IL-23 reverse active colitis in a T cell-mediated model of murine colitis [28].

The strong expression of IL-23 in ileal DCs in a mouse model and human CD [20], [22], [29] and the recent findings of the genome-wide association study [12] suggest that the IL-23/IL-23R cytokine receptor system is also involved in the pathogenesis of CD. Interestingly, IL-23 secretion in ileal DCs is driven by the intestinal bacterial microflora [30], and we recently demonstrated that ileal DCs may actively sample luminal bacteria via dendrites reaching the intestinal lumen [31]. However, only myeloid DCs and not plasmacytoid DCs secreted IL-23 upon stimulation with various toll-like receptor (TLR) ligands [32]. Increased IL-23 secretion has been shown in antigen-presenting cells (APCs) derived from lamina propria mononuclear cells of CD patients which could be down-regulated by anti-IL-12p40 mAb treatment [29]. In contrast, APCs from UC patients produced only baseline levels of IL-23 [29]. Moreover, a very recent study demonstrated in a mouse model that IL-23 producing DCs participate in intestinal granuloma formation [33] further pointing to a role for this cytokine in the pathogenesis of CD.

Since replication of genome-wide association findings in populations with different genetic background is essential to confirm disease susceptibility genes [34] and in order to clarify the phenotypic consequences of the different *IL23R* variants in IBD, we performed a detailed genotype-phenotype analysis in a large German IBD cohort (CD: n = 833; UC: n = 456; controls: n = 1289) by analyzing all 10 *IL-23R* variants for which significant linkage to CD has been demonstrated in the study by Duerr et al. [12]. In addition, we tested for epistasis of *IL23R* variants with *CARD15* gene variants [3], [4], [35], and SNPs in *SLC22A4* (solute carrier family 22, member 4), encoding the organic cation transporter 1 (OCTN1), and in *SLC22A5,* encoding OCTN2 [8], [10], [36]. Since these gene variants are proven CD susceptibility genes associated with distinct phenotypic characteristics [10], [37], [38], our aim was to investigate potential interactions with *IL23R* variants including epistatic effects on certain disease phenotypes. In a detailed genotype-phenotype analysis, we also analyzed the effects of *IL23R* variants on specific CD and UC disease subtypes.

## Methods

### Study population

The study population (n = 2670) was comprised of 1289 IBD patients of Caucasian origin including 833 patients with CD, 456 patients with UC, and 1381 healthy, unrelated controls. Written, informed consent was obtained from all patients. The study was approved by the local Ethics committee and adhered to the Ethical Principles for Medical Research Involving Human Subjects of the Helsinki Declaration (http://www.wma.net/e/policy/b3.htm). Demographic and clinical data (behaviour and location of IBD, disease-related complications, need for surgery or immunosuppressive therapy) were recorded by analysis of patient charts and a detailed questionnaire including an interview at time of enrolment. The demographic characteristics of the IBD study population were collected blind to the results of the genotype analyses and are summarized in Table 1. The diagnosis of CD or UC was determined according to established guidelines based on endoscopic, radiological, and histopathological criteria [39]. Patients with CD were assessed according to the Montreal classification [40] based on the age at diagnosis (A), location (L), and behaviour (B) of disease. In patients with UC, anatomic location was also based on the Montreal classification, using the criteria ulcerative proctitis (E1), left-sided UC (distal UC; E2), and extensive UC (pancolitis; E3). Patients with indeterminate colitis were excluded from the study.

**Table 1:** Demographic characteristics of the IBD study population.

	CD (n = 833)	UC (n = 456)
**Gender**		
Male (%)	46.0%	51.8%
Female (%)	54.0%	48.2%
**Age (yr)**		
Mean±SD	39.5±18.7	41.5±20.4
Range	10–80	7–85
**Age at diagnosis (yr)**		
Mean±SD	27.9±16.4	31.8±18.5
Range	7–71	9–81

### DNA extraction and genotyping of the IL23R variants

Genomic DNA was isolated from peripheral blood leukocytes by standard procedures using the DNA blood mini kit from Qiagen (Hilden, Germany). The 10 *IL23R* SNPs (rs1004819, rs7517847, rs10489629, rs2201841, rs11465804, rs11209026 = p.Arg381Gln, rs1343151, rs10889677, rs11209032, rs1495965), for which significant associations with CD were found in a previous study [12], were genotyped by PCR and melting curve analysis using a pair of fluorescence resonance energy transfer (FRET) probes in a LightCycler® 480 Instrument (Roche Diagnostics, Mannheim, Germany). The donor fluorescent molecule (fluorescein) at the 3′-end of the sensor probe is excited at its specific fluorescence excitation wavelength (533 nm) and the energy is transferred to the acceptor fluorescent molecule at the 5′-end of the anchor probe (LightCycler Red 610, 640, or 670). The specific fluorescence signal emitted by the acceptor molecule is detected by the LightCycler® 480 Instrument. The sensor probe matches exactly to one allele of each SNP, preferentially to the less common allele, whereas, in the case of the other allele, there is a mismatch resulting in a lower melting temperature. The total volume of the PCR was 5 µl containing 25 ng of genomic DNA, 1×Light Cycler 480 Genotyping Master (Roche Diagnostics), 2.5 pmol of each primer, and 0.75 pmol of each FRET probe (TIB MOLBIOL, Berlin, Germany). Amplification included an initial denaturation step (95°C for 10 min) followed by 45 cycles (95°C for 10 sec, primer annealing temperature as given in table S1 for 10 sec, 72°C for 15 sec). The melting curve analysis was comprised of an initial denaturation step (95°C for 1 min), a step rapidly lowering the temperature to 40°C and holding for 30 sec, and a heating step slowly increasing the temperature up to 80°C and continuously measuring the fluorescence intensity (1 acquisition/°C). The results of the melting curve analysis were confirmed by analyzing samples representing all possible genotypes using sequence analysis. All sequences of primers and FRET probes and primer annealing temperatures used for genotyping and for sequence analysis are given in the Supplementary data (Tables S1 and S2).

### Genotyping of the CARD15 and OCTN1/2 variants

Genotyping of the three common NOD2 variants p.Arg702Trp (rs2066844), p.Gly908Arg (rs2066847), and p.Leu1007fsX1008 (rs2066847) was performed as described previously [9]. In addition, the 1672 C→T SNP in *SLC22A4* (rs1050152) encoding OCTN1 and the –207 G→C SNP (rs2631367) in *SLC22A5* encoding OCTN2 were investigated by restriction fragment length polymorphism analysis as described previously [10] or by melting curve analysis using FRET probes (primer and probe sequences are available on request).

### Statistical analyses

Each genetic marker was tested for Hardy-Weinberg equilibrium in the control population. For comparison between categorical variables, Fisher's exact test or Χ^2^ test was used where appropriate. Single-marker allelic tests were performed with Pearson's Χ^2^ test. Student's t-test was applied for quantitative variables. All tests were two-tailed, considering p-values <0.05 as significant. Odds ratios were calculated for the minor allele at each SNP. For multiple comparisons, Bonferroni correction was applied. Data were evaluated by using the SPSS 13.0 software (SPSS Inc., Chicago, IL, USA) and R-2.4.1. (http://cran.r-project.org). Interactions between different polymorphisms were tested using logistic regression in R testing using the number of minor alleles as predictor variable thus implementing an Armitage test of trend.

## Results

### Frequency distribution of IL23R genotypes and their role in IBD susceptibility

In all three subgroups (CD, UC, and controls), the genotype frequencies of the 10 *IL23R* SNPs were in accordance with the Hardy-Weinberg equilibrium. All *IL23R* gene variants displayed highly significant associations with CD. The *P*-values ranged from 4.36×10^−5^ to 1.92×10^−11^ (Table 2). The distribution of wildtype, heterozygous and homozygous carriers of each *IL23R* variant in CD and UC compared to controls is given in Table S3. In CD, the strongest association was found for the SNP rs1004819 [*P* = 1.92×10^−11^; OR 1.56; 95 % CI (1.37–1.78)]. The allele frequencies for the SNP rs11209026 (p.Arg381Gln), which displayed the strongest association in the study of Duerr et al. [12], showed also highly significant differences between CD patients and controls in our study. The minor allele frequency was 0.030 in CD as compared to 0.068 in the controls [*P* = 8.04×10^−8^; OR 0.43; 95 % CI (0.31–0.59); Table 2], suggesting a protective effect against CD. By combining both SNPs in a logistic regression analysis, we were able to demonstrate independent effects on CD susceptibility. An additional independent effect could also be shown for rs7517847, while the other seven *IL23R* SNPs were not independently disease-associated.

**Table 2:** Associations of *IL23R* gene markers in CD and UC case-control association studies.

Gene marker	Minor allele	Crohn's disease n = 833			Ulcerative colitis n = 456			Controls n = 1381
		MAF	p value	OR [95 % CI]	MAF	p value	OR [95 % CI]	MAF
rs1004819	T	0.360	1.92×10^−11^	1.56 [1.37–1.78]	0.314	3.81×10^−3^	1.27 [1.08–1.50]	0.265
rs7517847	G	0.356	1.86×10^−9^	0.68 [0.60–0.77]	0.381	3.78×10^−4^	0.76 [0.65–0.88]	0.448
rs10489629	G	0.390	1.05×10^−6^	0.73 [0.65–0.83]	0.413	5.59×10^−3^	0.81 [0.69–0.94]	0.465
rs2201841	C	0.348	1.90×10^−8^	1.46 [1.28–1.66]	0.307	2.11×10^−2^	1.21 [1.03–1.43]	0.268
rs11465804	G	0.033	4.13×10^−5^	0.53 [0.39–0.72]	0.047	1.27×10^−1^	0.77 [0.54–1.08]	0.061
rs11209026	A	0.030	8.04×10^−8^	0.43 [0.31–0.59]	0.049	3.61×10^−2^	0.70 [0.50–0.98]	0.068
rs1343151	T	0.288	4.36×10^−5^	0.76 [0.67–0.87]	0.312	4.81×10^−2^	0.85 [0.72–1.00]	0.347
rs10889677	A	0.346	9.69×10^−9^	1.47 [1.29–1.68]	0.312	5.17×10^−3^	1.26 [1.07–1.49]	0.264
rs11209032	A	0.367	7.29×10^−8^	1.43 [1.25–1.62]	0.319	8.29×10^−2^	1.15 [0.98–1.36]	0.288
rs1495965	G	0.497	3.74×10^−6^	1.33 [1.18–1.51]	0.468	2.41×10^−2^	1.29 [1.02–1.38]	0.425

Minor allele frequencies (MAF), allelic test *P*-values, and odds ratios (OR, shown for the minor allele) with 95% confidence intervals (CI) are depicted for both the CD and UC case-control cohorts.

In contrast to CD, the associations of *IL23R* SNPs with susceptibility to UC were markedly weaker. However, with the exception of rs11465804 and rs11209032, all other *IL23R* variants were significantly associated with UC in the univariate analysis (Table 2). For these eight SNPs, the odds ratios differentiating between protection against UC (OR<1.0) or increased UC susceptibility (OR>1.0), were very similar to those observed for CD (Table 2), suggesting overall similar disease-modifying effects of the different *IL23R* variants on CD and UC. The most strongly UC-associated SNP was rs7517847 [*P* = 3.78×10^−4^; OR = 0.76; 95 % CI (0.65–0.88)]. Similar to CD, the frequency of the SNP rs11209026 (p.Arg381Gln) was significantly decreased in UC patients compared to the controls, although this association was much weaker than in CD [*P* = 3.61×10^−2^; OR = 0.70; 95 % CI (0.50–0.98)] in the univariate analysis (Table 2). While the minor allele of these two *IL23R* variants protected against UC, the minor allele of rs1004819 was associated with UC in the univariate analysis [*P* = 3.81×10^−3^; OR = 1.27; 95 % CI (1.08–1.50)]. Comparing only SNPs with ORs>1.0 in this analysis, rs1004819 was the most strongly UC-associated *IL23R* variant, similar to our results in CD. However, stepwise forward logistic regression analysis revealed only rs7517847 as an independent risk factor for UC.

### Associations between IL23R genotype and IBD phenotype

Having confirmed that *IL23R* is an IBD susceptibility gene also in the German population, we next investigated potential phenotypic consequences of *IL23R* variants in a well-characterized subgroup of patients from the Munich IBD center. Table 3 summarizes the disease characteristics of CD patients depending on the rs11209026 (p.Arg381Gln) *IL23R* genotype status. In CD patients with different rs11209026 (p.Arg381Gln) genotypes, there were no differences in the frequency of disease characteristics such as age of onset, disease behaviour, disease location as defined by the Montreal classification [40], extraintestinal involvement, and other complications of CD such as incidence of stenoses, fistulas, abscess formation, or the use of immunosuppressive agents (Table 3), although there was a trend towards less surgical interventions in carriers of the A allele of the p.Arg381Gln variant, which, however, did not reach statistical significance [*P* = 0.067; OR 0.39; CI (0.15–1.05)]. A subgroup analysis based on the type of surgery (e.g., stricturoplasty, ileocecal resection, fistulectomy, colectomy) did not demonstrate significant associations to a certain type of surgical intervention nor to a certain cause of surgery, e.g., ileal stenosis (data not shown). However, this analysis was limited by the fact that the subgroup analyzed for phenotypic consequences did not contain AA homozygous carriers of the rs11209026 (p.Arg381Gln) variant.

**Table 3:** Association between *IL23R* p.Arg381Gln (rs11209026) genotype and CD disease characteristics in the subcohort of the Munich IBD center (n = 452) for which detailed phenotypic data based on the Montreal classification [40] were available.

*IL23R* p.Arg381Gln (rs11209026) genotype status	(1) GG	(2) GA*	(1) vs. (2) p value	(1) vs. (2) OR (95% CI)
*(n = 452)*	*(n = 430)*	*(n = 22)*		
**Male sex** *(n = 449)*	46.1%	45.2%	1.000	0.97 (0.52–1.81)
**Age at diagnosis (yr)** *(n = 449)*				
Mean±SD	27.8±16.3	31.6±18.2	0.207	N/A
Range	7–71	12–56		
**Disease duration (yr)** *(n = 444)*				
Mean±SD	12.2±7.7	9.4±6.6	0.200	N/A
Range	0–44	3–32		
**Age at diagnosis** *(n = 444)*				
≤16 years (A1)	12.1%	4.5%	0.495	0.35 (0.05–2.63)
17–40 years (A2)	74.9%	72.7%	0.804	0.90 (0.34–2.34)
>40 years (A3)	13.0%	22.7%	0.200	1.96 (0.70–5.53)
**Location** *(n = 449)*				
Terminal ileum (L1)	12.9%	13.6%	1.000	1.07 (0.31–3.73)
Colon (L2)	17.1%	27.3%	0.248	1.82 (0.69–4.80)
Ileocolon (L3)	69.3%	59.1%	0.347	0.64 (0.27–1.53)
Upper GI (L4)	0.7%	0.0%	1.000	N/A
**Behaviour** 1 *(n = 447)*				
Non-stricturing, Non-penetrat. (B1)	21.6%	27.3%	0.596	1.36 (0.52–3.57)
Stricturing (B2)	25.6%	13.6%	0.312	0.46 (0.13–1.58)
Penetrating (B3)	52.7%	59.1%	0.663	1.30 (0.54–3.10)
**Use of immunosuppressive agents** 2 *(n = 286)*	83.6%	70.6%	0.183	0.47 (0.16–1.40)
**Surgery because of CD** 3 *(n = 439)*	52.0%	30.0%	0.067	0.39 (0.15–1.05)
**Fistulas** *(n = 450)*	52.3%	59.1%	0.663	1.31 (0.55–3.14)
**Stenosis** *(n = 452)*	63.0%	54.5%	0.499	0.70 (0.30–1.67)

For each variable, the number of patients included is given.

* In this subgroup, there were no AA homozygous carriers for the genotype-phenotype analysis available.

1 Disease behaviour was defined according to the Montreal classification [40]. A stricturing disease phenotype was defined as presence of stenosis without penetrating disease. The diagnosis of stenosis was made surgically, endoscopically, or radiologically (using MRI enteroclysis).

2 Immunosuppressive agents included azathioprine, 6-mercaptopurine, 6-thioguanin, methotrexate, and/or infliximab.

3 Only surgery related to CD-specific problems (e.g. fistulectomy, colectomy, ileostomy) was included.

Analyzing the phenotypic contribution of the rs1004819 *IL23R* variant, which had overall the most significant association with CD, there was an increased incidence of ileal involvement in TT homozygous carriers (93.2%) compared to CC wildtype carriers (78.0%) [*P* = 0.004; OR 4.24; CI (1.46–12.34); Table 4] which, however, lost significance after Bonferroni correction. Similarly, there was an increased incidence of stenosis in rs1004819 TT homozygotes compared to carriers of the CC genotype [(74.6% vs. 59.6%; P = 0.045; OR 1.99; CI (1.04–3.82); Table 4] which also did not fulfill the criteria of significance after Bonferroni correction. Moreover, the variant rs7517847, for which we demonstrated an association with UC in the stepwise logistic regression analysis, was not associated with any specific disease subtype (data not shown).

**Table 4:** Associations between *IL23R* SNP rs1004819 genotypes and CD disease characteristics in the subcohort of the Munich IBD center (n = 457) for which detailed phenotypic data based on the Montreal classification [40] were available.

*IL23R* rs1004819 genotype status	(1) CC	(2) CT	(3) TT	(1) vs. (2) p value	(1) vs. (3) p value	(2) vs. (3) p value
*(n = 457)*	*(n = 193)*	*(n = 205)*	*(n = 59)*			
**Male sex** *(n = 457)*	48.7%	45.9%	39.0%	0.616	0.233	0.375
**Age at Diagnosis (yr)** *(n = 413)*						
Mean±SD	27.3±12.5	28.5±14.5	28.1±15.8	0.297	0.697	0.833
Range	11–65	11–71	7–71			
**Disease duration (yr)** *(n = 413)*						
Mean±SD	11.9±8.6	12.0±8.6	13.4±10.7	0.889	0.476	0.515
Range	2–35	0–33	3–44			
**Age at diagnosis** *(n = 413)*						
≤16 years (A1)	12.8%	8.3%	13.5%	0.175	1.000	0.284
17–40 years (A2)	76.7%	76.2%	71.2%	1.000	0.465	0.460
>40 years (A3)	10.6%	15.5%	15.4%	0.211	0.334	1.000
**Location** *(n = 451)*						
Terminal ileum (L1)	12.0%	13.4%	13.6%	0.762	0.821	1.000
Colon (L2)	21.5%	16.4%	6.8%	0.245	**0.011,OR 0.27 CI 0.09–0.78**	0.080
Ileocolon (L3)	66.0%	69.2%	79.7%	0.519	0.053	0.140
Upper GI (L4)	0.5%	1.0%	0.0%	1.000	1.000	1.000
Any ileal involvement (L1+L3)	76.4%	82.6%	93.2%	0.135	**0.004,OR 4.24 CI 1.46–12.34**	0.060
**Behaviour** ^1 ^ *(n = 452)*						
Non-stricturing, Non-penetrat. (B1)	22.5%	21.3%	18.6%	0.808	0.591	0.719
Stricturing (B2)	26.2%	22.3%	35.6%	0.410	0.187	**0.043, OR 1.93 CI 1.03–3.61**
Penetrating (B3)	51.3%	56.4%	45.8%	0.313	0.552	0.181
**Surgery because of CD** ^3 ^ *(n = 441)*	48.1%	50.3%	62.7%	0.683	0.054	0.103
**Fistulas** *(n = 455)*	50.8%	56.3%	45.8%	0.314	0.553	0.183
**Stenosis** *(n = 457)*	59.6%	59.6%	74.6%	0.607	**0.045,OR 1.99 CI 1.04–3.82**	0.090

Significant *P*-values are given in bold letters including corresponding odds ratios (OR) and 95% confidence intervals (CI). However, none of these *P*-values remained significant after Bonferroni correction (*P*>0.05). For each variable, the number of patients with a complete data set is given. Footnotes: See legend of Table 3 for detailed explanation.

### Analysis for epistasis between IL23R, CARD15 and SLC22A4/5 genes in CD patients

Finally, we analyzed potential evidence for epistasis of *IL23R* variants with the other three replicated IBD susceptibility genes *CARD15* [3], [4], [35], *SLC22A4,* and *SLC22A5* [8], [10], [36] including their effects on overall disease susceptibility and phenotype. In all three subgroups (CD, UC, and controls), the frequencies of the analyzed NOD2 variants (p.Arg702Trp, p.Gly908Arg, p.Leu1007fsX1008) and of the SNPs in *SLC22A4* (1672 C→T) and in *SLC22A5* (–207 G→C) were in accordance with the Hardy-Weinberg equilibrium. Except from *SLC22A5* (–207 G→C), all SNPs were highly associated with CD but not with UC (Table 5). No evidence for epistasis between these three CD susceptibility genes and *IL23R* variants was found in CD and UC**.** None of the *P*-values found was significant after correction for multiple testing; the lowest *P*-value for CD was 0.147 for the interaction between rs11209026 and NOD2 mutation status with an associated OR of 1.33 [CI (0.92–1.91)]. There was evidence for epistatic gene-gene interactions regarding several phenotypic disease characteristics which are summarized in the Supplementary Data (Table S4 and S5). However, given the large number of interactions analyzed (n = 364), none of these *P* values remained significant at a *P* level of <0.05 after Bonferrroni correction.

**Table 5:** Association of *CARD15*, *SLC22A4* and *SLC22A5* variants with CD (n = 833) and UC (n = 456) compared to the control population (n = 1381).

SNP	P value	OR	95% CI
**Crohn's disease** (n = 833)			
CARD15 R702W	4.02×10^−5^	1.92	1.39–2.64
CARD15 G908R	9.43×10^−3^	1.81	1.14–2.87
CARD15 1007fs	2.75×10^−15^	3.68	2.54–5.35
Any CARD15 variant	2.41×10^−15^	2.89	2.15–3.63
*SLC22A4* (1672 C→T)	1.23×10^−2^	1.15	1.03–1.28
*SLC22A5* (–207 G→C)	7.71×10^−2^	0.87	0.74–1.02
**Ulcerative colitis** (n = 456)			
CARD15 R702W	0.932	1.02	0.66–1.58
CARD15 G908R	0.704	0.89	0.47–1.66
CARD15 1007fs	0.174	0.64	0.34–1.23
Any CARD15 variant	0.320	0.83	0.58–1.19
*SLC22A4* (1672 C→T)	0.573	1.04	0.91–1.18
*SLC22A5* (–207 G→C)	0.976	1.00	0.83–1.21

OR: odds ration; 95% CI: 95% confidence interval.

## Discussion

The present study confirms the previously described association of *IL23R* gene variants with CD in a large German IBD patient cohort and demonstrates a particular role for the rs1004819 variant in the pathogenesis of CD in German patients. It is the first study analyzing for potential epistasis between *IL23R* variants and the three other previously described CD susceptibility genes *CARD15, SLC22A4,* and *SLC22A5*. Our results also verify the previously identified role of rs11209026 (p.Arg381Gln) as a protective variant in CD [12]. The greatest influence on the disease risk for CD was seen in homozygous carriers of the minor allele of the rs1004819 and rs11209026 (p.Arg381Gln) gene variants with an odds ratio of 2.46 and 0.41, respectively, compared to healthy controls, demonstrating opposing effects of these two SNPs on CD susceptibility. By confirming the results obtained by Duerr et al. [12], our study supports the validity of chip-based technologies for the rapid identification of disease susceptibility genes in genome-wide association studies.

The identification of rs1004819 as an independent major CD susceptibility variant in our patient cohort contrasts with the data published by Duerr et al. [12] and those of a related study with an overlapping North American study population [41], in which rs7517847 had the strongest association to CD. In a very recent British study, in contrast, the strongest association to CD was found for rs11209026 (p.Arg381Gln) [13], while another genome-wide association study from Belgium identified (in addition to rs11209026) rs11465804 as the strongest CD-associated marker [42]. Overall, these studies confirm *IL23R* as a CD susceptibility gene, but demonstrate differences for certain *IL23R* variants regarding the strength of their disease-modifying effect in different populations.

Importantly, our study also revealed similar, but markedly weaker associations of several *IL23R* SNPs with UC, suggesting that certain *IL23R* variants may also contribute to the pathogenesis of UC which is not the case for the other main CD susceptibility gene *CARD15*. As these SNPs were not completely consistent to those found in the previous genome-wide study [12], it has to be acknowledged that the present study included only cases with sporadic UC, whereas Duerr et al. [12] investigated familial UC.

A detailed genotype-phenotype analysis revealed weak associations of the *IL23R* rs1004819 variant with ileal involvement (*P* = 0.004) and stenoses (*P* = 0.045) in carriers of the TT genotype compared to carriers of the CC wildtype genotype. However, both associations did not fulfill significance criteria after Bonferroni correction. Nevertheless, the prevalence of ileal involvement in carriers of the TT genotype of the rs1004819 variant (93.2%) was remarkable high. Given that the original study by Duerr et al. [12] analyzed only ileal cases of CD, it has been assumed that some of the *IL23R* variants may predispose to an ileal disease phenotype. However, a very recent British study could not identify a certain disease phenotype associated with *IL23R* variants but the strength of the association between CD and the rs1004819 variant was almost 1000fold weaker than in our study group (*P* = 1.1×10^−8^ vs. 1.92×10^−11^) [13] which may partly explain the different results of these two genotype-phenotype analyses. It has to be acknowledged that our genotype-phenotype analysis was limited to patients with detailed phenotype status available which were all part of the Munich IBD center patient population. Moreover, none of the patients included in the phenotype analysis was homozygous for the AA genotype of the rs11209026 variant. Therefore, it can not be excluded that larger multicenter trials or metaanalyses may reveal a certain disease phenotype associated with *IL23R* variants.

Interestingly, *IL23R* was also identified as a disease-associated gene in other chronic inflammatory diseases. The rs10489629 variant was found to be associated with chronic periodontitis (Glas et al., unpublished data), while in psoriasis, a predisposing haplotype including the p.Arg381Gln variant (rs11209026) has been described [43]. In both CD [44] and psoriasis [45], treatment with an anti-p40 IL-12/23 antibody demonstrated therapeutic efficacy. Therefore, *IL23R* potentially represents a susceptibility gene and therapeutic target in various chronic inflammatory diseases. However, the degree and the variety of associated SNPs may differ in the different diseases. Possibly, *IL23R* variants result in alternative mRNA splicing, leading to corresponding IL-23R isoforms [46] with different tissue distributions. Functional studies will be necessary to clarify this issue.

Supporting the results of the first pilot study in human CD [44], neutralizing antibodies to the p40 subunit, which block both IL-12 and IL-23, suppressed established chronic intestinal inflammation in an animal model of CD [47], [48]. Studies in knockout mice and the efficacy of antibodies against the IL-23-specific p19 subunit suggest that IL-23 rather than IL-12 drives chronic intestinal inflammation [23], [28]. The T-cell response activated by IL-23 is attenuated by SOCS3, which acts as a feedback inhibitor to regulate Th17 cells [49]. Interestingly, we recently demonstrated activation of STAT3 by IL-22 [50], [51], which is also produced in Th17 cells [52], and attenuation of IL-22 signaling by SOCS3 overexpression [51]. Although selective targeting of IL-23 is now emerging as an attractive concept, p19-deficient mice are highly susceptible to T cell–mediated colitis [22].

Despite the successful proof of principle for the applicability of genome-wide association studies in the genetics of complex diseases, the contribution of certain *IL23R* variants to CD or UC is not sufficiently strong to explain the genetic background of IBD on its own. Other candidate genes are likely to be involved and recent genome wide scans suggest also *ATG16L1* [41], [42], [53], *PHOX2B*, *NCF4*, *FAM92B* [41], and a region modulating *PTGER4* expression [42] as CD susceptibility genes, but further confirmatory studies are necessary. In contrast, a multitude of studies demonstrated that certain NOD2 variants, particularly p.Arg702Trp, p.Gly908Arg, and p.Leu1007fsX1008, are associated with CD, but not with UC. We and others demonstrated a strong association with an ileal disease phenotype particularly for the p.Leu1007fsX1008 variant [37], [38]. Interestingly, in this study, p.Leu1007fsX1008 was the most strongly CD-associated gene variant in a combined multivariate analysis (Supplementary Data, Table S6). The other CD susceptibility gene repeatedly confirmed is located within the IBD5 locus and most likely represented by certain OCTN variants [8], [10]. We therefore also investigated possible interactions of *IL23R* variants with these IBD susceptibility genes. We could not demonstrate an epistatic interaction between *IL23R* variants and the analyzed variants in the *SLC22A4, SLC22A5* and *CARD15* genes on IBD susceptibility but demonstrated for some gene variants an epistatic influence on certain disease characteristics. However, these interactions lost significance after Bonferroni correction.

In conclusion, we confirmed the association of *IL23R* with IBD. In addition to the protective *IL23R* variant rs11209026 (p.Arg381Gln), we identified rs1004819 as independent risk factor for developing CD and UC in the German population which may also contribute to an ileal disease phenotype in CD. A combined analysis of variants in three other CD susceptibility genes (*CARD15, SLC22A4, SLC22A5*) found no evidence for epistasis to *IL23R* regarding CD susceptibility.

## Supporting Information

Table S1 (0.04 MB DOC)

Table S2 (0.03 MB DOC)

Table S3 (0.07 MB DOC)

Table S4 (0.04 MB DOC)

Table S5 (0.04 MB DOC)

Table S6 (0.03 MB DOC)
