# Supplementary material for: rs1004819 Is the Main Disease-Associated IL23R Variant in German Crohn's Disease Patients: Combined Analysis of IL23R, CARD15, and OCTN1/2 Variants
Source: PLoS One. 2007 Sep 5;2(9):e819. doi: 10.1371/journal.pone.0000819 (PMC1950565; doi:10.1371/journal.pone.0000819)
Supplement: Table S1 — (0.04 MB DOC) [file pone.0000819.s001.doc]

| **Polymorphism** | **Primer sequences** | **Primer annealing** | **FRET probe sequences** |
| --- | --- | --- | --- |
| rs1004819 | TTCTAGGACCCTTTTGGC  TTTGCAAAAATATGAACTCATTCAA | 58°C | AGATAGCACAGTAAGAATCACAGC-FL  LC610-AAGCAGGTCAGTGCAATCCAGATT |
| rs7517847 | CTGCCAATTCCCTAAACA  GACAGCCCATAAAGATACAAACA | 59°C | AAGGCCTCAGCTACACCTGTAT-FL  LC670-GCTAGAAACTGGAGCCCTCCCAACTC |
| rs10489629 | AGGTGTCATTACCCACCAGCA  CTGCCTAGCAAGATTATGCAA | 58°C | GTCAGCCACATTTGGTAGTG-FL  LC640-TTAGCGCTACTTTACAAAAGCGGAAAC |
| rs2201841 | AGGAATGTAGGCAGCCTCTAG  GTGCTGGGCTTACAGGCAG | 59°C | TATAGAAGATGATGACCTCAAGAAA-FL  LC640-GCATAATCATAGGCCAGGCATGGTG |
| rs11465804 | CACATGGAATTCTGGGCTA  AAGGCATATCTTATTGTCCAGAAA | 57°C | GTAGTCTTTTAGTAATTGCC-FL  LC610-ACCCATCATACTGAAAAAATCACATCAGG |
| rs11209026 | CTTTCCTTTCATTAGACAACAGAGG  AACTGAAATGACTAAATTTTGGTGA | 56°C | ACAGATCATTCCAAACTGGGT-FL  LC640-GTTTTTGCAGAATTTCTGTTTTCTGATTT |
| rs1343151 | CGAAAGAAAGATTATTTCATGAAGC  AGTGTGTCCAGTTGCTCAC | 57°C | TTGAATGTTCTTTGCCAAATTG-FL  LC670-CACTGAGTTTCTCCTTCTTGCCATCA |
| rs10889677 | TCCATGCCTTTTTAATTTTAGC  GACTCTATAAAAAATACATGAGGCGTC | 59°C | TTCTGCCTCATTTCTTAAAATTAGA-FL  LC610-TAAGGTCCCGAAGGTGGAACATGC |
| rs11209032 | GGTGTTTGTTTTATCTTGTACGC  CAATCCGGTGGTTCTTCACAG | 59°C | CTTTGCAATGGCAGATGGAAG-FL  LC640-TGGCAATAAATGCAATTCAGCTTGAAG |
| rs1495965 | CAGGAATAAATGTGAATGAGAACAGAC  CAATATTTATATCTCCCATGGCTC | 58°C | GAAAATGTTCTCTTCCTCCACAG-FL  LC670-GGATTAACATTTGCTTCTGGCAGC |

**Supplementary data, Table S1.** Primer sequences, FRET probe sequences, and primer annealing temperatures used for genotyping *IL23R* variants.

Note: FL: Fluorescein, LC610: LightCycler-Red 610; LC640: LightCycler-Red 640; LC670: LightCycler-Red 670. The polymorphic position within the sensor probe is underlined. A phosphate is linked to the 3'-end of the anchor probe to prevent elongation by the DNA polymerase in the PCR.
