# Supplementary material for: rs1004819 Is the Main Disease-Associated IL23R Variant in German Crohn's Disease Patients: Combined Analysis of IL23R, CARD15, and OCTN1/2 Variants
Source: PLoS One. 2007 Sep 5;2(9):e819. doi: 10.1371/journal.pone.0000819 (PMC1950565; doi:10.1371/journal.pone.0000819)
Supplement: Table S2 — (0.03 MB DOC) [file pone.0000819.s002.doc]

| **Polymorphism** | **Primer sequences** | **Primer annealing** |
| --- | --- | --- |
| rs1004819 | AGCACCCTCAAGGCCAT  ATTCAACATCTGAGTCTTGTGTAAC | 60°C |
| rs7517847 | CTGCCAATTCCCTAAACA  GACAGCCCATAAAGATACAAACA | 55°C |
| rs10489629 | AGGTGTCATTACCCACCAGCA  CTGCCTAGCAAGATTATGCAA | 55°C |
| rs2201841 | TGGATTTCTCACCTCCAGAAC  GTGCTGGGCTTACAGGCAG | 50°C |
| rs11465804 | CACATGGAATTCTGGGCTA  AAGGCATATCTTATTGTCCAGAAA | 55°C |
| rs11209026 | CTTTCCTTTCATTAGACAACAGAGG  AGAGTTTGGCATGGGTAAGTAC | 50°C |
| rs1343151 | GTTTGCCTTCCTTACAAGGGTA  GAAAAGCAAGACCCTTGGAT | 55°C |
| rs10889677 | AGTTCTACCAATCTTGTTTCCAG  GACTCTATAAAAAATACATGAGGCGTC | 60°C |
| rs11209032 | GGTGTTTGTTTTATCTTGTACGC  CTTCCAAATAGGATGCACAATGAG | 55°C |
| rs1495965 | CAGGAATAAATGTGAATGAGAACAGAC  TCTTTCCAGTTAGGGGTCAA | 60°C |

**Supplementary data, Table S2.** Primer sequences and annealing temperatures used for the sequence analysis of *IL23R* variants.
