# Supplementary material for: rs1004819 Is the Main Disease-Associated IL23R Variant in German Crohn's Disease Patients: Combined Analysis of IL23R, CARD15, and OCTN1/2 Variants
Source: PLoS One. 2007 Sep 5;2(9):e819. doi: 10.1371/journal.pone.0000819 (PMC1950565; doi:10.1371/journal.pone.0000819)
Supplement: Table S3 — (0.07 MB DOC) [file pone.0000819.s003.doc]

| **Polymorphism** | **Genotype** | **Crohn’s disease**  n=833 | | **Ulcerative colitis**  n=456 | | **Controls**  n=1381 |
| --- | --- | --- | --- | --- | --- | --- |
|  |  | **Allele frequency** | **p value** | **Allele frequency** | **p value** | **Allele frequency** |
| rs1004819 | CC | 0.409 | 2.35 x 10^-10^ | 0.457 | 5.14 x 10^-3^ | 0.540 |
|  | CT | 0.461 |  | 0.457 |  | 0.390 |
|  | TT | 0.130 |  | 0.086 |  | 0.070 |
| rs7517847 | TT | 0.403 | 4.92 x 10^-9^ | 0.365 | 5.39 x 10^-4^ | 0.305 |
|  | TG | 0.482 |  | 0.509 |  | 0.494 |
|  | GG | 0.115 |  | 0.126 |  | 0.201 |
| rs10489629 | AA | 0.378 | 7.69 x 10^-6^ | 0.318 | 1.53 x 10^-3^ | 0.288 |
|  | AG | 0.465 |  | 0.539 |  | 0.492 |
|  | GG | 0.157 |  | 0.143 |  | 0.220 |
| rs2201841 | TT | 0.432 | 2.31 x 10^-7^ | 0.463 | 2.19 x 10^-2^ | 0.540 |
|  | TC | 0.440 |  | 0.460 |  | 0.385 |
|  | CC | 0.128 |  | 0.077 |  | 0.075 |
| rs11465804 | TT | 0.936 | 1.96 x 10^-5^ | 0.910 | 6.02 x 10^-2^ | 0.881 |
|  | TG | 0.061 |  | 0.086 |  | 0.117 |
|  | GG | 0.003 |  | 0.004 |  | 0.002 |
| rs11209026 | GG | 0.940 | 4.95 x 10^-8^ | 0.907 | 3.85 x 10^-2^ | 0.866 |
|  | GA | 0.059 |  | 0.089 |  | 0.131 |
|  | AA | 0.001 |  | 0.004 |  | 0.003 |
| rs1343151 | CC | 0.513 | 1.21 x 10^-4^ | 0.469 | 1.40 x 10^-1^ | 0.421 |
|  | CT | 0.399 |  | 0.438 |  | 0.463 |
|  | TT | 0.088 |  | 0.093 |  | 0.116 |
| rs10889677 | GG | 0.435 | 1.13 x 10^-7^ | 0.455 | 6.27 x 10^-3^ | 0.544 |
|  | GA | 0.439 |  | 0.466 |  | 0.384 |
|  | AA | 0.126 |  | 0.079 |  | 0.072 |
| rs11209032 | GG | 0.404 | 7.21 x 10^-7^ | 0.451 | 1.05 x 10^-1^ | 0.510 |
|  | GA | 0.459 |  | 0.460 |  | 0.404 |
|  | AA | 0.137 |  | 0.089 |  | 0.086 |
| rs1495965 | AA | 0.257 | 2.30 x 10^-5^ | 0.259 | 1.45 x 10^-2^ | 0.330 |
|  | AG | 0.493 |  | 0.545 |  | 0.490 |
|  | GG | 0.250 |  | 0.196 |  | 0.180 |

**Supplementary Data, Table S3.** Distribution of wildtype, heterozygous and homozygous carriers of each *IL23R* variant in CD and UC compared to controls. *P* values are given for the comparison of genotype frequencies in cases vs. controls.
