# Supplementary material for: rs1004819 Is the Main Disease-Associated IL23R Variant in German Crohn's Disease Patients: Combined Analysis of IL23R, CARD15, and OCTN1/2 Variants
Source: PLoS One. 2007 Sep 5;2(9):e819. doi: 10.1371/journal.pone.0000819 (PMC1950565; doi:10.1371/journal.pone.0000819)
Supplement: Table S4 — (0.04 MB DOC) [file pone.0000819.s004.doc]

| **Disease characteristic** | **SNP1** | **SNP2** | **Marginal P SNP1** | **Marginal P SNP2** | **Interaction** |
| --- | --- | --- | --- | --- | --- |
| Upper GI tract involvement (L4) | CARD15 R702W | *IL23R* rs11465804 | 0.5384 | 0.7539 | 0.0048 |
| Upper GI tract involvement (L4) | CARD15 R702W | *IL23R* rs11209026 | 0.5329 | 0.5628 | 0.0037 |
| Fistulas | CARD15 R702W | *IL23R* rs7517847 | 0.5237 | 0.7640 | 0.0094 |
| Stenoses | CARD15 1007fs | *IL23R* rs11465804 | 0.0021 | 0.3354 | 0.0077 |
| Stenoses | *IL23R* rs1343151 | *IL23R* rs1495965 | 0.2191 | 0.1270 | 0.0094 |
| Abscess | *IL23R* rs2201841 | *IL23R* rs10889677 | 0.1869 | 0.3238 | 0.0062 |
| Use of infliximab | *IL23R* rs10489629 | *IL23R* rs11209032 | 0.0753 | 0.1257 | 0.0060 |

**Supplementary Data, Table S4.** Epistatic interactions between *CARD15* and *IL23R* variants for certain phenotypic disease characteristics in CD with a significance level of *P* < 0.01. However, given the large number of interactions analyzed (n=364), none of these *P* values remained significant at a *P* level of < 0.05 after Bonferrroni correction.
