# Supplementary material for: rs1004819 Is the Main Disease-Associated IL23R Variant in German Crohn's Disease Patients: Combined Analysis of IL23R, CARD15, and OCTN1/2 Variants
Source: PLoS One. 2007 Sep 5;2(9):e819. doi: 10.1371/journal.pone.0000819 (PMC1950565; doi:10.1371/journal.pone.0000819)
Supplement: Table S5 — (0.04 MB DOC) [file pone.0000819.s005.doc]

| **Disease characteristic** | **SNP1** | **SNP2** | **Marginal P SNP1** | **Marginal P SNP2** | **Interaction** |
| --- | --- | --- | --- | --- | --- |
| Left-sided UC (E2) | CARD15 R702W | *IL23R* rs1004819 | 0.0884 | 0.6095 | 0.0046 |
| Left-sided UC (E2) | CARD15 R702W | *IL23R* rs11209032 | 0.1158 | 0.8497 | 0.0063 |
| Pancolitis (E3) | CARD15 1007fs | *IL23R* rs1004819 | 0.4352 | 0.4541 | 0.0070 |
| Pancolitis (E3) | CARD15 1007fs | *IL23R* rs1343151 | 0.4151 | 0.6076 | 0.0013 |
| Pancolitis (E3) | CARD15 1007fs | *IL23R* rs11209032 | 0.4553 | 0.8354 | 0.0046 |
| Extraintestinal manifestations | CARD15 variants (R702W, G908R, 1007fs combined) | *IL23R* rs10489629 | 0.7311 | 0.5265 | 0.0086 |

**Supplementary Data, Table S5.** Epistatic interactions between *CARD15* and *IL23R* variants for certain phenotypic disease characteristics in UC with a significance level of *P* < 0.01. However, after Bonferrroni correction, none of these interactions remained significant at a *P* level of < 0.05.
