# Supplementary material for: rs1004819 Is the Main Disease-Associated IL23R Variant in German Crohn's Disease Patients: Combined Analysis of IL23R, CARD15, and OCTN1/2 Variants
Source: PLoS One. 2007 Sep 5;2(9):e819. doi: 10.1371/journal.pone.0000819 (PMC1950565; doi:10.1371/journal.pone.0000819)
Supplement: Table S6 — (0.03 MB DOC) [file pone.0000819.s006.doc]

| **Disease** | **SNP** | **P value** | **OR** | **95% CI** |
| --- | --- | --- | --- | --- |
|  |  |  |  |  |
| CD | *IL23R* rs1004819 | 3.51 x 10^-5^ | 1.50 | 1.24-1.82 |
| CD | *IL23R* rs7517847 | 6.20 x 10^-4^ | 0.73 | 0.61-0.88 |
| CD | *IL23R* rs11209026 | 1.08 x 10^-4^ | 0.47 | 0.32-0.69 |
| CD | CARD15 R702W | 1.38 x 10^-6^ | 2.21 | 1.60-3.04 |
| CD | CARD15 1007fs | 1.41 x 10^-13^ | 4.03 | 2.79-5.83 |
|  |  |  |  |  |
| UC | *IL23R* rs7517847 | 1.07 x 10^-3^ | 0.72 | 0.59-0.88 |

**Supplementary Data, Table S6.** Multivariate analysis regarding CD and UC susceptibility of *IL23R*, *CARD15*, *SLC22A4* and *SLC22A5* gene variants. A total of 15 polymorphisms, including 10 *IL23R* SNPs, 3 *CARD15* SNPs (R702W, G908R, 1007fs), *SLC22A4* (1672 C→T) and *SLC22A5* (–207 G→C), were included into this analysis. Multiple logistic regression analysis was performed with a stepwise forward procedure applying an inclusion criterion of 0.0033 (equal to 0.05/15). SPSS 15.0 was used for this analysis. OR: odds ratio; 95% CI: 95% confidence interval; CD: Crohn’s disease; UC: ulcerative colitis.
